# Advantages of digital technology in the assessment of bone marrow involvement in Gaucher's disease

**DOI:** 10.3389/fmed.2023.1098472

**Published:** 2023-05-12

**Authors:** Esther Valero-Tena, Mercedes Roca-Espiau, Jose Verdú-Díaz, Jordi Diaz-Manera, Marcio Andrade-Campos, Pilar Giraldo

**Affiliations:** ^1^Departamento de Medicina Interna y Reumatología, Hospital MAZ, Zaragoza, Spain; ^2^Fundación Española para el Estudio y Terapéutica de la Enfermedad de Gaucher y otras Lisosomales (FEETEG), Zaragoza, Spain; ^3^John Walton Muscular Dystrophy Research Center, Newcastle University, Newcastle upon Tyne, United Kingdom; ^4^Grupo Español de Enfermedades de Depósito Lisosomal de la SEHH (GEEDL), Madrid, Spain; ^5^Grupo de Investigación en Hematología, Instituto de Investigación Hospital del Mar, IMIM-Parc de Salut Mar, Barcelona, Spain

**Keywords:** Gaucher disease, bone marrow MRI, bone disease, random forest machine-learning study, predictive factors

## Abstract

Gaucher disease (GD) is a genetic lysosomal disorder characterized by high bone marrow (BM) involvement and skeletal complications. The pathophysiology of these complications is not fully elucidated. Magnetic resonance imaging (MRI) is the gold standard to evaluate BM. This study aimed to apply machine-learning techniques in a cohort of Spanish GD patients by a structured bone marrow MRI reporting model at diagnosis and follow-up to predict the evolution of the bone disease. In total, 441 digitalized MRI studies from 131 patients (M: 69, F:62) were reevaluated by a blinded expert radiologist who applied a structured report template. The studies were classified into categories carried out at different stages as follows: A: baseline; B: between 1 and 4 y of follow-up; C: between 5 and 9 y; and D: after 10 years of follow-up. Demographics, genetics, biomarkers, clinical data, and cumulative years of therapy were included in the model. At the baseline study, the mean age was 37.3 years (1–80), and the median Spanish MRI score (S-MRI) was 8.40 (male patients: 9.10 vs. female patients: 7.71) (*p* < 0.001). BM clearance was faster and deeper in women during follow-up. Genotypes that do not include the c.1226A>G variant have a higher degree of infiltration and complications (*p* = 0.017). A random forest machine-learning model identified that BM infiltration degree, age at the start of therapy, and femur infiltration were the most important factors to predict the risk and severity of the bone disease. In conclusion, a structured bone marrow MRI reporting in GD is useful to standardize the collected data and facilitate clinical management and academic collaboration. Artificial intelligence methods applied to these studies can help to predict bone disease complications.

## Introduction

Type 1 Gaucher disease (GD1) (OMIM#230800) is an autosomal recessive lysosomal storage disorder due to deficient activity of acid beta-glucocerebrosidase (GBA), which results in intracellular accumulation of glucosylceramide (GluCer) primarily within cells of the mononuclear phagocyte system. GD is caused by variants in the *GBA1* gene. GluCer accumulation is multisystemic mainly in the liver and spleen, with musculoskeletal involvement being common and leading to complications that compromise normal physical activity (1).

Glycosylsphingolipid accumulation in bone marrow compromises normal hematopoietic function, mainly for the platelet series, and cytokine release, and the underlying inflammatory component cause intraosseous ischemic events (1–3). The bone marrow effect in Gaucher disease has been described as infiltration by Gaucher cells in bone marrow (bone marrow burden) and other manifestations, such as the ischemic vascular events and their sequelae, namely bone infarcts (diaphysis), osteonecrosis (joint surface), osteolysis, osteosclerosis, and joint damage (3). In pediatric age groups, growth retardation and altered bone remodeling lead to decreased bone mineral density. Bone manifestations are one of the most serious complications of GD with a prevalence of ~80% (3, 4), and they are associated with physical disability and reduced quality of life (5). The pathophysiology of vascular obstruction is not fully elucidated; recently, immune phenomena and angiogenesis imbalance have been described (6, 7).

The bone marrow is an extensive organ that is difficult to be evaluated by conventional imaging methods; however, magnetic resonance imaging (MRI) has proved to be a useful tool for obtaining a global map of the contents of the medullary cavity. MRI can distinguish differences and abnormalities by visualizing the balance between the fat and the medullary hematopoietic cellular component, providing an image of the variations that occur between these components within the bone cavity (8). The assessment of bone marrow involvement is often complex due to the presence of multiple patterns and the evolutionary change of these over the course of life stages, gender, and disease progression. In addition, the bone marrow is an organ that can be affected by different diseases, such as hematological neoplasms, metastases, or genetic entities, such as lysosomal disorders (9).

In GD, an MRI helps in the assessment of BM infiltration patterns and the detection of complications, such as bone crises, infarcts, necrosis, and fractures. It must be considered that infiltration in GD occurs centrifugally, starting from the spine and spreading to the limbs, while the process of BM infiltration clearance during therapy occurs in the opposite direction, although complications, such as infarcts, avascular necrosis, and vertebral fractures, are irreversible lesions (10).

Some semi-quantitative scales have been described based on the analysis of BM infiltration signal alterations detected as MRI patterns at different locations. Both the bone marrow burden (BMB) (11) and the Spanish MRI (S-MRI) scores are used for initial and therapy response assessment (12, 13). S-MRI is more extensive and includes analysis of bone marrow infiltration in vertebral bodies, pelvis, and femur; also, it quantifies the presence of complications, such as necrosis, infarcts, bone crises, and vertebral fractures (10, 12).

Nowadays, structured report forms have gained importance among radiologists to standardized reports according to organ and/or diseases (14, 15). MRI is the gold standard for bone marrow assessment; however, interpretation and reporting vary among centers and difficult collaborations. Our group has recently published a structured report based on eight items (demographic data, diagnostic suspicion, technical data, type of exam, initial or control, patterns and involvement distribution, complications and their location, and summarized comments). It has been designed to provide guidance for radiologists when reporting S-MRI protocol assessments to unified criteria, allow comparisons, and decrease interobservers' variability (16).

Machine learning is revolutionizing the way data are analyzed in clinics and is helping to develop digital tools for diagnosis, disease progression prediction, and treatment responses. In the case of rare diseases, where clinicians often have limited clinical experience, these tools can be especially useful to speed the diagnosis and obtain better prognosis assessments and personalized care.

This study aimed to apply a structured bone marrow MRI reporting model in a cohort of Spanish GD patients at diagnosis and follow-up and to use machine-learning techniques to predict the evolution of the bone disease.

## Patients and methods

### Study design

A retrospective study of MRI scans performed at diagnosis and follow-up in the Unit of Lysosomal Disorders and evaluated by the Spanish Group of GD from April 1995 to May 2022 was conducted. A total of 441 bone marrow MRI examinations (S-MRI protocol) were included from 131 patients diagnosed with GD. Infiltration in the lumbar spine, pelvis, and femora was evaluated, according to the signal intensity on T1 and T2 WI. Progressive values for each MRI pattern were assigned following S-MRI description: homogeneous (H, 4 points), non-homogeneous diffuse (NHD, 3 points), non-homogeneous mottled (NHM, 2 points), or non-homogeneous reticular (NHR,1 point), and normal or no infiltration (N,0) (12). The existence of complications (infarcts, necrosis, fractures, arthropathy, or bone crisis) was also taken into account (4 points). We applied the structured bone marrow MRI report in each imaging study (16). The report model and S-MRI description are included in Supplementary Figure 1.

All studies were reevaluated by the same expert radiologist in a blinded fashion to ensure that the structured report model and analysis were conducted as objectively as possible.

We also analyzed the information reported in each patient's clinical record, collecting demographic, genetic, clinical, and analytical data; the type of treatment; and the accumulated years of treatment exposure. The variables analyzed are listed in Supplementary Table 1.

To assess the evolution of bone involvement over time, the studies were divided into four groups. The first group (group A) included studies performed at diagnosis or before starting therapy (baseline). The second group included studies performed between the 1st and 4th year of follow-up (group B). The third group included studies performed between the 5th and 9th year of follow-up (group C), and the fourth group included studies performed after 10 years or more of follow-up (group D).

As part of the evaluation of bone involvement, bone mineral density was also estimated considering the criteria recommended by the WHO for the diagnosis of osteopenia and osteoporosis. The T-score or Z-score was used accordingly (17). Gender, genotype, spleen status, type of therapy, age at the start of treatment, and accumulated years of treatment in different subgroups were also considered.

### Statistical analysis

A descriptive study was carried out, presenting qualitative variables as percentages. Mean and standard deviation were presented for those quantitative variables that followed a normal distribution, and median, interquartile range, or range between maximums and minimums were presented for those that did not follow a normal distribution. In addition, a correlation analysis was undertaken between numerical variables using Pearson's linear correlation index, and for categorical variables, the X^2^ test was performed.

The data were analyzed using the IBM SPSS statistics 27.0 statistical program.

For the comparative analysis of the results, the Student's *t*-test for the comparison of independent samples was used for quantitative variables. To determine the suitability of this test, an analysis of the normality of the distributions was first performed.

### Machine learning

From multiple random forest test models, three were selected and trained to identify features that can predict the risk of bone complications. Bone complications were defined by the presence of intraosseous ischemic events (bone crisis, infarcts, avascular necrosis, and fractures) during the follow-up. Model A included all variables that were described as significant in a previously published study from our group using the same population (18), model B considered whether a treatment was applied or not, and model C ignored the S-MRI punctuation score.

The model parameters were optimized using a grid search. As random forest modeling uses bootstrapping, no cross-validation was used to reduce the training time. Model performance on the validation dataset was evaluated using the ROC curve, AUC, accuracy, and f1-score. The model was created using the scikit-learn package for Python 3.10.4 (19).

## Results

### General characteristics

The cohort included 131 GD patients (62 female patients and 69 male patients) and 310 follow-up bone marrow MRI studies. The general characteristics of the patients are detailed in Table 1.

**Table 1:** General characteristics at baseline.

	**Males (*n* = 69)**	**Females (*n* = 62)**	**Total (*n* = 131) (range)**
Age at baseline (years)	37.49 ± 16.45	44.15 ± 17.47	37.31 (12–53)
Mean age at diagnosis (years)	21.78 ± 15.54	26.77 ± 17.19	24.63 (1–65)
Mean age at the start of therapy (years)	30.71 ± 17.17	31.15 ± 18.43	31.0 (1–47)
Accumulated years on therapy	6.91 ± 6.30	7.81 ± 6.45	7.39 (2–18)
Median S-MRI at baseline	9.10 ± 6.36	7.71 ± 4.65	8.4 (0-20)
Spleen removal *n* (%)	20 (28.9)	11 (17.4)	31 (23.8)
*GBA1*(NM_000157.4) GD# *n* (%)			131
[c.1226A>G]+[c.1226A>G]	4 (5.8)	9 (14.5)	13 (10.0)
[c.1226A>G]+[c.1448T>C]	22 (31.9)^b^	22 (35.5)^b^	44 (33.6)
[c.1226A>G]+[other]	31 (44.9)^b^	26 (41.9)^b^	57 (43.5)
[other]+[other]	12 (17.4)	5 (8.0)	17 (12.9)
BMD at baseline, *n* (%)			119
normal	30 (52.6)	36 (58.0)	66 (55.5)
osteopenia	15 (26.3)	10 (16.1)	25 (21.0)
osteoporosis	12 (21.0)	16 (25.8)	28 (23.5)
Patients without therapy, *n* (%)	6 (8.7)	11(17.7)	17 (13.0)
Patients under therapy*	63 (91.3)	51 (82.2)	114 (87.0)

#c.1226A>G (N370S), c.1448T>C (L444P).

Mean ± SD; S-MRI, Spanish MRI score; BMD, bone mineral density.

^*^Number of patients exposed to any therapy at any time during follow-up (ERT: 210; miglustat: 49; eliglustat: 23).

### Bone disease by MRI

**Figure 1:**
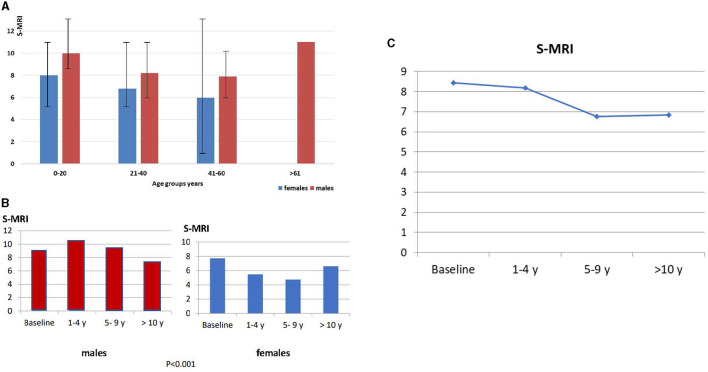
**(A)** S-MRI distribution according to age group and gender. **(B)** S-MRI distribution in relation to sex. **(B)** At baseline, S-MRI in male patients is 9.1 vs. S-MRI in female patients is 7.7 (*p* < 0.001). After 10 years of follow-up, the mean S-MRI in male patients is 7.0 and mean S-MRI in female patients is 4.7 after 5–9 years of follow-up. **(C)** Globally, the S-MRI is 8.4, and the maximum reduction is observed after 5–9 years on therapy.

At the time of the first MRI, the median S-MRI was 8.4 (95% CI 0–25) (Figure 1C). The median S-MRI at baseline according to age groups was years: 0–20: 8.78; 21–40: 7.78; 41–60: 7.68; >61: 11.0; S-MRI according to gender distribution and age group is shown in Figure 1A.

Regarding gender distribution, the median S-MRI at baseline was higher in male patients [9.10 (95% CI 0–25)] than in female patients [7.71 (95% CI 0–24)], and this difference was significant (*p* < 0.001). During the follow-up, the reduction in bone marrow infiltration was different between men and women. A 20.8 % decrease in S-MRI was observed in male patients after 10 years on therapy, while female patients achieved a 39.0% reduction in S-MRI earlier (5–9 years) (Figure 1B). No significant differences in genotype distribution, age, and spleen status were observed between genders (see Table 1). Globally, the maximum reduction was observed after 5–9 years on therapy, and it remained stable after 10 years of follow-up (Figure 1C).

**Figure 2:**
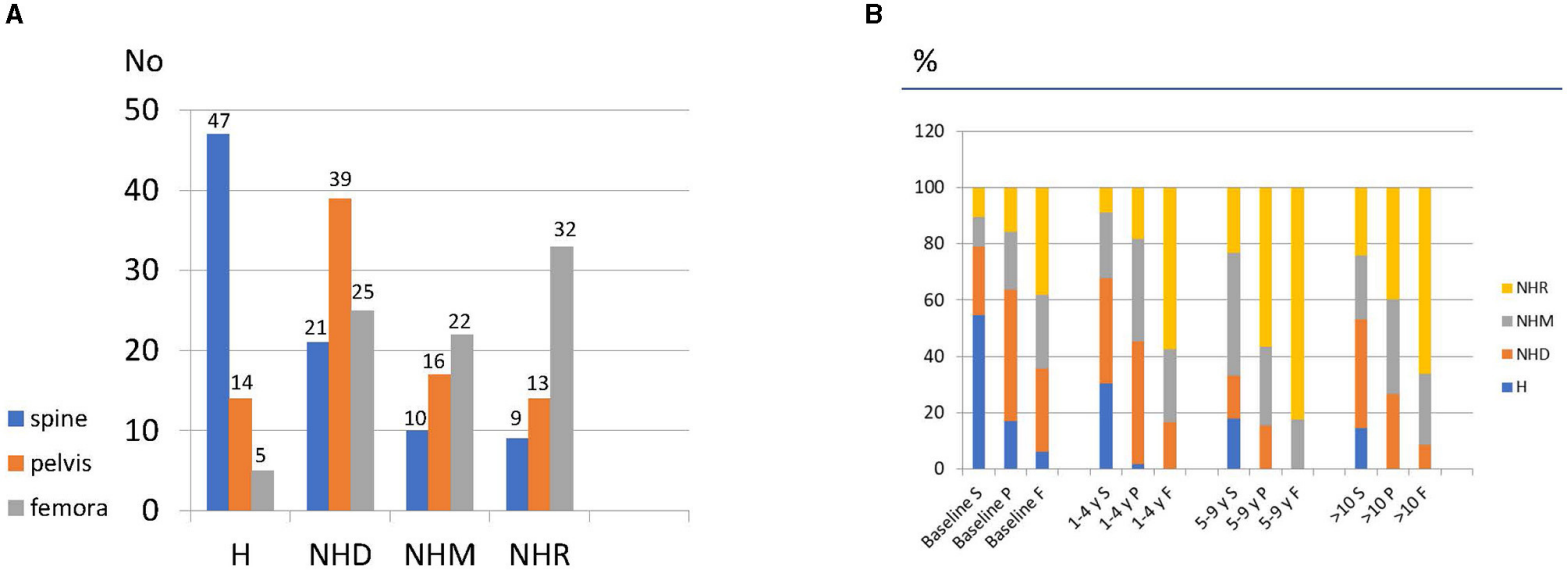
**(A)** Distribution of infiltration patterns in bone marrow at baseline according to location and **(B)** percentage of changes in the infiltration pattern at follow-up.

Bone marrow infiltration in all locations was found in 80 (61.5%) patients: 38 (47.5%) were female patients, and 42 (52.5%) were male patients. The MRI pattern was homogeneous in the lumbar spine in 47 (36.1%) patients, in the pelvis in 14 (10.7%) patients, and in the femurs in 5 (3.8%) patients (Figure 2A). At baseline, 63 patients (48.4%) showed complications such as bone crisis, fractures, AVN, or infarcts.

According to genotypes, patients were classified into homozygous c.1226A>G (N370S/N370S), heterozygous c.1226A>G/c.1448T>C (N370S/L444P), heterozygous c.1226A>G/other variant, and other different variants (Table 1). The analysis at baseline of the degree of BM infiltration and bone complications according to genotype showed no significant differences between 13 homozygous c.1226A>G (N370S/N370S) and 44 heterozygous c.1226A>G/c.1448T>C (N370S/L444P), but a significant difference between the 57 heterozygous c.1226A>G/other and 17 other/other genotype patients with different variants (*p* = 0.017) was observed (see Supplementary Figure 2).

### MRI changes during follow-up

There were 310 follow-up studies of these patients available for evaluation, which were analyzed according to the specified time groups. The patterns reported at baseline in the different locations (S: spine; P: pelvis; and F: femur) are represented with color code in Figure 2A. The changes (%) in the infiltration pattern during follow-up at each site using the same color code according to the groups were A (baseline), B (1–4 years follow-up), C (5–9 years follow-up), and D (10 or more years follow-up) (Figure 2B).

The analysis of changes in infiltration patterns related to the type of treatment was difficult due to the diversity of treatments used over time and the diversity of applied doses of enzyme replacement therapy. To obtain some useful information, we classified the patients generically in enzyme replacement therapy (ERT) distributed in their follow-up groups and we differentiated between patients treated with miglustat and those treated with eliglustat (Table 1).

### Bone disease and spleen status

The median S-MRI in 31 patients with spleen removal at baseline was 13.16 (95% CI 0–25), which was significantly higher than that in non-splenectomized patients (6.96, 95% CI 0–24) (*p* < 0.001). At follow-up, the differences persisted with a median S-MRI in group D (10 or more years) in non-splenectomized patients of 5.42 (95% CI 3.8–6.3) vs. 9.02 (95% CI 4.5–12.7) in splenectomized patients (*p* < 0.001). Patients with spleen removal also had a significantly higher incidence of bone complications (40.8 vs. 16.0%; *p* 0.0001). No patients were splenectomized during follow-up.

### Bone mineral density

Bone mineral density was evaluated in 119 (90.8%) patients following the WHO criteria using the Tor Z-score when applicable (15). In the first study, osteoporosis was defined in 28 (23.5%), osteopenia in 25 (21.0%), and was normal in the rest (66; 55.5%). A decrease in BMD was observed in 74.6% of splenectomized patients compared to 43.8% of non-splenectomized patients (*p* < 0.001).

Bone mineral density data were available from 256 follow-up studies, of which 170 corresponded to patients under 50 years of age and 86 to those over 51 years old. In terms of gender, 45.0% of male patients and 56.4% of female patients under 50 years presented a decrease in BMD, and this difference was not significant (*p* = 0.4). In the group over 51 years of age, there was a decrease in bone mineral density of 44.0% in male patients and 61.6% in female patients; this difference was significant (*p* = 0.04). Analysis of the follow-up of BMD in the different time periods showed no changes in the male patients, while in female patients there was a significant increase in the loss of BMD throughout the follow-up period in this study (*p* = 0.001).

There was no evidence of a relationship between decreased BMD and the presence of intraosseous vascular events during follow-up.

Analysis of the *GBA1* genotype within the different subgroups showed that patients homozygous for c.1226A>G (N370S) had a significantly lower prevalence of lost BMD compared to the rest of the genetic subgroups (*p* = 0.003).

### Machine learning

**Figure 3:**
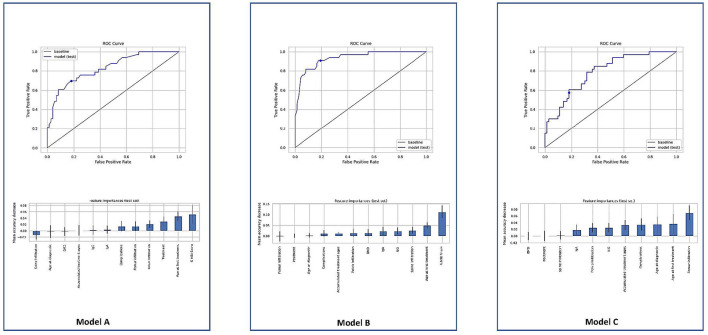
ROC models. The point marks the decision boundary threshold of 0.5. A model includes all variables described as significant in a previous study. ROC B model considers whether any treatment was applied or not and features the importance of model B using the mean decrease in accuracy. ROC C model does not contain the S-MRI score and had a substantial drop in accuracy of 74.29% and an f1-score of 69.92%.

The random forest model A achieved an AUC of 75.82% with an accuracy of 78.10% and an f1-score of 75.18%. We obtained a true positive rate (TPR) of 69.70% and a false positive rate (FPR) of 18.06% with a decision boundary threshold of 0.5 (Figure 3A). Using the mean decrease in accuracy as the feature importance metric, the most important features for this model were the S-MRI, the age at first treatment, and the treatment used. The random forest model B, which considered whether a treatment was applied or not, achieved an AUC of 85.73% with an accuracy of 83.81% and an f1-score of 87.21%. We obtained a true positive rate (TPR) of 90.91% and a false positive rate (FPR) of 19.44% with a decision boundary threshold of 0.5 (Figure 3B). Using the mean decrease in accuracy as the feature importance metric, the most important features for this model were the S-MRI, the age at first treatment, and the extent of spine infiltration.

In order to understand the importance of each variable in the developed model, new models were generated removing one single variable at a time. The variable with more relevance in the accuracy was the S-MRI. Model C did not contain the S-MRI score and had a substantial drop in accuracy; it achieved an AUC of 69.76% with an accuracy of 74.29% and an f1-score of 69.92%. We obtained a true positive rate (TPR) of 57.58% and a false positive rate (FPR) of 18.06% with a decision boundary threshold of 0.5 (Figure 3C). Using the mean decrease in accuracy as the feature importance metric, the most important features for this model were the femur infiltration, age at first treatment, and the diagnostic age.

## Discussion

### Bone disease

For almost 30 years, GD patients have been treated with ERT, which is effective in clearing substrate deposits, especially at the visceral level. However, bone involvement and its complications (bone infarcts, avascular necrosis, fractures, bone crisis, cortical thinning, osteopenia, and osteoporosis or lytic bone lesions) are problems that have not been completely solved with current treatments (2, 20, 21). The progressive storage of glucocerebroside in the bone marrow, cytokine imbalances, and vascular compromise are some of the proposed explanations for the development of bone complications, although there is no clear explanation about why it appears in some patients and does not in others with similar clinical and genetic characteristics (22). Bone complications are the major cause of morbidity and one of the most debilitating aspects of GD (1, 3, 21–23). Despite various efforts to study different biomarkers to predict the development and intensity of bone involvement and its complications, no specific markers have been identified (24–28).

### MRI

At baseline, our results are in accordance with previous publications that show severe bone involvement in splenectomized patients (18, 29). In addition, these patients had a significant loss of BMD, which may increase the risk of fractures in this subgroup of patients although this estimate should be taken with caution as there may be a bias in the estimation of BMD due to the presence of infarcts, especially in the femoral neck (30). Interestingly, there was a significant difference in the S-MRI at baseline between male (9.10) and female patients (7.70) (*p* = 0.047), which was not previously reported. This could be justified in part by the differences in bone maturation according to sex (31), which have been reported in MRI imaging performed in healthy subjects, mainly in the lumbar spine. MRI imaging showed that male and female subjects convert hematopoietic marrow to fatty marrow in the lumbar vertebral bodies in significantly different ways (32). Nevertheless, other factors, such as hormonal or vascular changes, may be involved. In addition, clinical characteristics (spleen status, genotype, and age) may have some significance; however, in our analysis, non-significance was found. Other studies were carried out with different methodologies such as the Dixon quantitative chemical shift imaging (QCSI reported by M Maas et al. to assess bone marrow in the spine of healthy subjects) (33). This technique allows assessing the fat fraction of the bone marrow with greater accuracy applied to a normal population stratified by age/gender; the results are in agreement with those previously reported by Ishijima et al. (33). The fat fractions in female subjects slowly increases with age until 44 years and rapidly after 45 years. In males, there is a rapid increase in the fat fraction up to the age of 25 years and it stabilizes in the following decades up to the age of 60 years. More recently, another tool has been described to measure the fat fraction in the lumbar spine. Fat fraction quantification of the bone marrow in the lumbar spine using the LiverLab assessment tool with results superimposable to those obtained using QCSI (34).

Our follow-up data show that the maximum reduction in BM infiltration occurs between 5 and 9 years on therapy, subsequent studies showed stability, and this is in line with other groups' observations (35). As expected, low baseline infiltration in women was associated with faster clearance.

No significant differences were identified regarding the age at diagnosis, age at first therapy, and the number of complications between male and female patients. However, the differences according to gender persisted during follow-up, with a median S-MRI after 10 or more years of 7.29 in male patients vs. 6.60 in female patients (*p* < 0.001), this was independent of bone mineral density status (1, 10, 18).

There is enormous variability in bone marrow patterns between age groups; some conditions, such as post-bleeding anemia or therapies, may complicate bone marrow image interpretation (36–38). In any case, training is required and uniformity in the description of its assessment by the radiologist is desirable (12). The application of the structured report template in our cohort for BM MRI studies improved standardization and the quality of the radiology reports, allowing easy comparison and the incorporation of data into the machine-learning system.

### Machine learning

In the area of rare diseases, the use of machine learning provides an opportunity to analyze agglomerated and heterogeneous data to create quality predictive models and identify risk features (39). This can be useful to improve the study of small cohorts of patients (40–42) and facilitate differential diagnosis; recently, its application in neuromuscular diseases was reported (43).

In line with our previous study (18), the best-generated model (model A) identified the S-MRI score and the age at first treatment as risk predictors for developing advanced bone disease. In this model, femur and pelvis infiltration at baseline and homogeneous pattern infiltration at any location also predicted a greater degree of bone involvement. It is logical that infiltration in locations, such as the pelvis and femur, is related to the extent of the disease. In addition, as expected, the intensity of the infiltration pattern (homogeneous > non-homogeneous) had an impact on the severity of bone involvement. These aspects are not considered independently at the initial calculation of the overall staging of the disease (5) but they are included in the S-MRI score (10, 12). However, they could be potential independent risk features for developing advanced bone disease and support the decision of early treatment (44). Further validation in another cohort is warranted.

We have observed that S-MRI is consistently the most important variable across all the models developed. Age and treatment contributed to the final accuracy indeed but to a lower extent, and their contribution is not consistent in all of the developed models. Model A was demonstrably the best of the three and includes information about all the different therapies. Model A could be therefore implemented into a webpage algorithm or a service for clinicians to feed it with the data and obtain a potential prediction of the prognosis of the patient.

In addition, this study supports the importance of early diagnosis to allow for tailored therapy. Delays in the diagnosis and therapy were related to bone complications in this cohort.

## Conclusion

- Our series is characterized by the homogeneity of the MRI studies and patient follow-up time. The greater bone involvement observed in men and the faster clearance of deposits in the bone detected in women stand out, without a clear explanation.
- The application of machine-learning models identified that the extent of the infiltration in MRI studies and the infiltrative pattern were able to translate the severity of the bone disease.
- Femur infiltration and a homogeneous infiltration pattern are predictive of the severity of the bone disease and could be potential independent risk factors.
- The study and the predictive model need to be validated in other series of patients to corroborate and extend the findings.

## Data availability statement

The original contributions presented in the study are included in the article/Supplementary material, further inquiries can be directed to the corresponding author.

## Ethics statement

The studies involving human participants were reviewed and approved by CEICA. Written informed consent to participate in this study was provided by the participants' legal guardian/next of kin.

## Author contributions

PG, MA-C, and EV-T: conception and design. JV-D and JD-M: machine-learning study and interpretation. EV-T: administrative support. PG and MR-E: provision of study materials or patients. EV-T, PG, JV-D, and JD-M: collection and assembly of data. All authors: data analysis and interpretation, manuscript writing, and final approval of the manuscript.
