# Supplementary figures and images for: Advantages of digital technology in the assessment of bone marrow involvement in Gaucher's disease
Source: Front Med (Lausanne). 2023 May 12;10:1098472. doi: 10.3389/fmed.2023.1098472 (PMC10213682; doi:10.3389/fmed.2023.1098472)

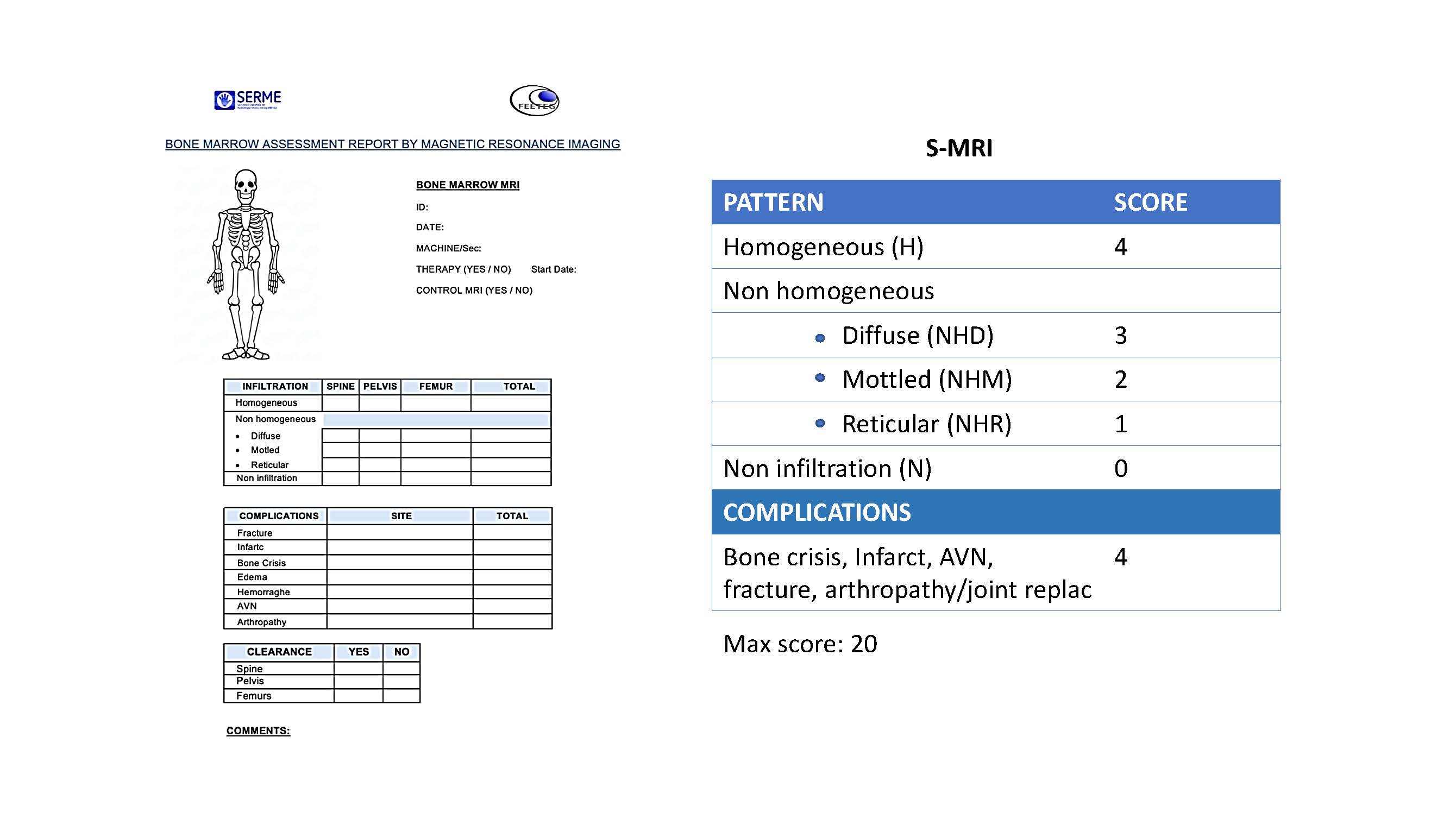

Supplement: Supplementary file 1 [file Image_1.JPEG]

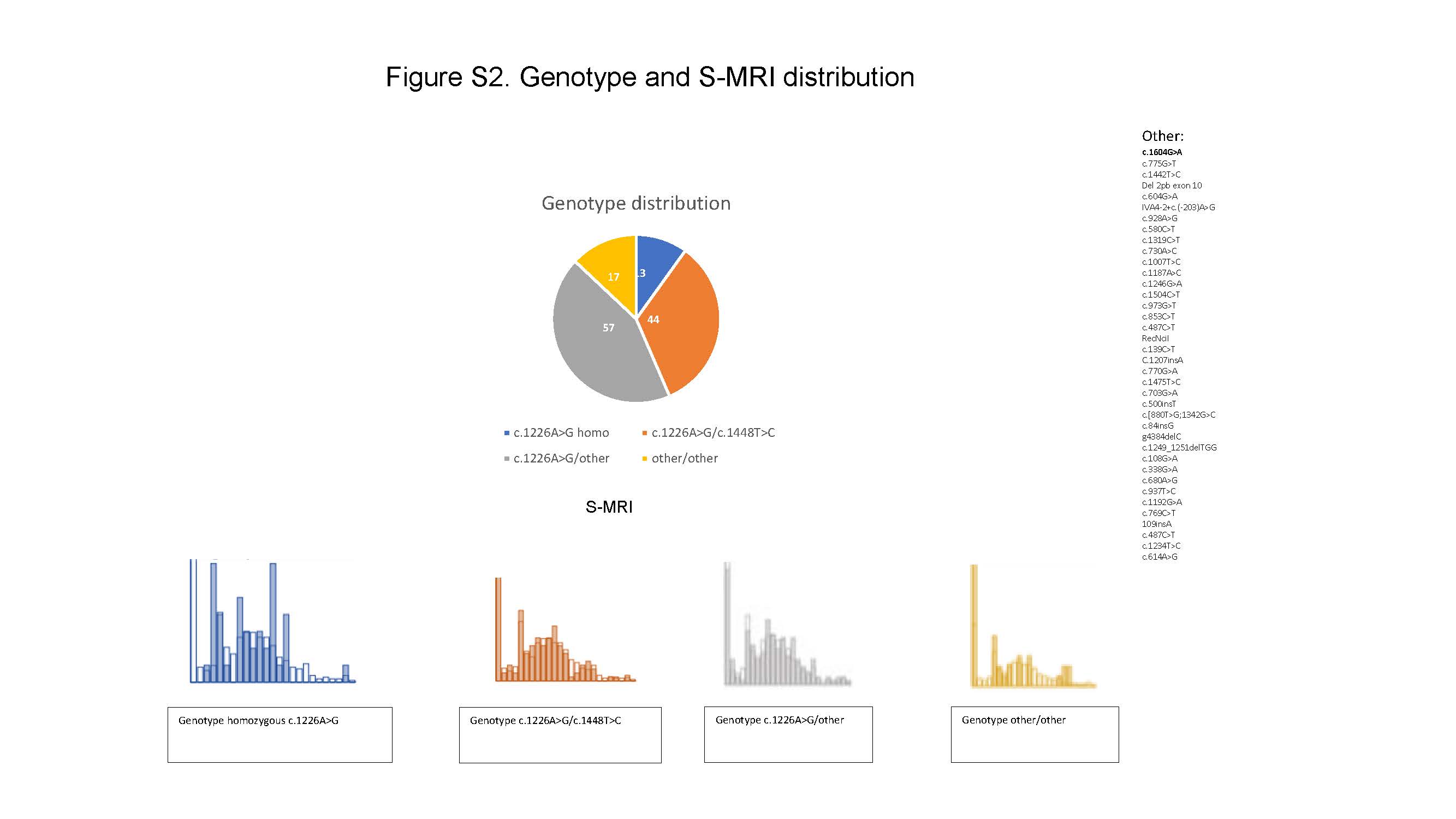

Supplement: Supplementary file 2 [file Image_2.JPEG]
