# Supplementary material for: Advantages of digital technology in the assessment of bone marrow involvement in Gaucher's disease
Source: Front Med (Lausanne). 2023 May 12;10:1098472. doi: 10.3389/fmed.2023.1098472 (PMC10213682; doi:10.3389/fmed.2023.1098472)
Supplement: Supplementary file 3 [file Table_1.docx]

Supplementary material

Table S1. Description of study variables

| **Demographics** |  |
| --- | --- |
| Gender | M/F |
| Birthdate | dd/mmm/year |
| Age at diagnosis | years |
| Cosanguinity | Y/N |
| Family history of PD | Y/N |
| Death date | Y/N |
| Survival | years |
| **Clinical Data** |  |
| GD-DS3 | mild  moderate  severe |
| Spleen removal | Y/N |
| Liver volume | MN |
| Spleen volume | MN |
| Previous bone crisis | Y/N |
| **Image Data** |  |
| S-MRI |  |
| DEXA | Z score  T score |
| **Analytical Data** |  |
| Hemoglobin | g/dL |
| WBC | x109/L |
| Platelets | x109/L |
| B12 vitamin level -serum concentrations, | pg/mL |
| Iron concentration | mg/dL |
| Cholesterol | mg/dL |
| Triglycerides | mg/dL |
| HDL-cholesterol | mg/dL |
| LDL-cholesterol | mg/dL |
| AST/ALT | UI |
| GGT/ alkaline phosphatase | UI |
| Bilirrubin | mg/dL |
| IgG-, IgA-, IgM | mg/dL |
| **Diagnosis** |  |
| GCase activity | nmol/mL/h |
| *GBA1* genotype | NM_000157 |
| **Biomarkers** |  |
| ChT | nmol/mL/h |
| *CHIT1* genotype NM_0003465 | Homozygous  Heterozygous  N |
| CCL18/PARC | ng/mL |
| GluSph | ng/mL |
| Ferritin | mcg/L |
| **Follow-up** | (5-25 y) |
| Age at start of therapy | years |
| Type of therapy | ERT  SRT  N |
| New bone crisis | Y/N |
| Joint replacement | Y/N |
| Neoplasia | Y/N |
| PD | Y/N |
| Other comorbidities | Y/N |
